# Rare documented case report of a retroperitoneal hernia as a complication to an anterior retroperitoneal spinal exposure

**DOI:** 10.1016/j.ijscr.2018.10.059

**Published:** 2018-10-29

**Authors:** Christina Colosimo, Naveed Ismail, Jonathan Schoeff, Chris Geiger, David Lundy

**Affiliations:** Sky Ridge Medical Center, Office of Graduate Medical Education, 10101 RidgeGate Pkwy, Lone Tree, CO, 80124, United States

**Keywords:** Anterior retroperitoneal spinal exposure, Retroperitoneal hernia, Small bowel obstruction, Internal hernia, Peritoneal disruption

## Abstract

**Introduction:**

Anterior retroperitoneal spinal exposures are widely used today for spinal surgeries. Incisional hernias are a documented complication of anterior spine exposures; however, there are no documented cases of hernias into the dissected retroperitoneal space.

We presented this exceptionally rare patient's complication to underscores the critical importance of preserving the peritoneum as a biologic barrier during retroperitoneal spine exposures.

**Presentation of case:**

An obese 54 year-old female with a history of two recurrent small bowel obstructions treated conservatively after a retroperitoneal spinal exposure presented with another small bowel obstruction. The patient was taken to the operating room for exploratory laparotomy. Intraoperatively, the patient was found to have multiple loops of small bowel herniated through a small defect in the peritoneum. The small bowel was severely adherent to the retroperitoneum, resulting in torsion and obstruction.

**Discussion:**

Based on intraoperative findings, we feel that the retroperitoneal hernia was directly related to the anterior lumbar spine exposure, in which peritoneal disruption is a well-reported phenomenon. There was mention of a peritoneal defect noted during the spine exposure procedure, with attempts to primarily close the defect.

**Conclusion:**

While disruption of the peritoneum occurs not infrequently during these primarily retroperitoneal procedures, this case should serve as a cautionary tale and reinforce the need for identification and immediate repair of any peritoneal defects that may be created during this type of procedure.

## Highlights

• Anterior exposures advantages including restoration of disk height, reduction of anterolisthesis, and coronal and sagittal balance.
• Incisional hernias are a documented complication of anterior exposures; however, there are no documented cases of retroperitoneal hernias.
• This case underscores the critical import of preserving the peritoneum as a biologic barrier during retroperitoneal spine exposures.

## 1 Introduction

Anterior retroperitoneal spinal exposures are widely used today for spinal surgeries; the anterior approach has several distinct advantages over the posterior approach including restoration of disk height and lumbar lordosis, reduction of anterolisthesis, and achievement of coronal and sagittal balance [1]. Documented complications include major vascular injury, prolonged ileus, wound related complications, deep venous thrombosis, somatic neural injury, bowel injury and autonomic dysfunction, which may lead to retrograde ejaculation in male [2]. Incidence remains quite low 2.7% [3] for major vascular injuries requiring repair; however, the implications of these complications can be profoundly devastating with 0.3% [4] death for anterior approaches. Incisional hernias are a documented complication of anterior spine exposures as well; however, there are no documented cases of hernias into the dissected retroperitoneal space.

Here we present a case of a recurrent small bowel obstruction secondary to adhesions between small bowel and exposed retroperitoneal structures including the left psoas muscle, resulting from a herniation of small bowel through a peritoneal defect apparently created at the time of an anterior retroperitoneal lumbar spinal exposure. This patient was managed in a community hospital. This work has been reported in line with the SCARE criteria [5].

## 2 Presentation of case

**Fig. 1:**
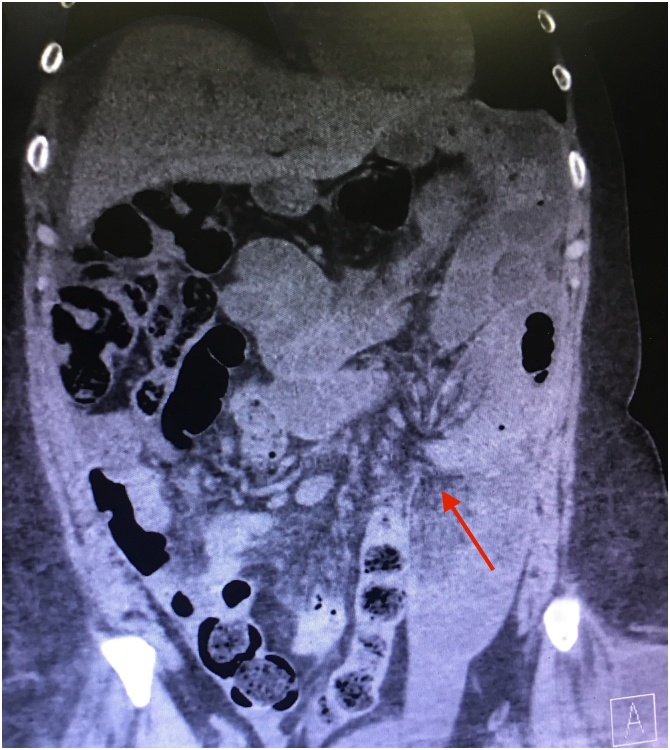
Computed tomography (CT) image of the retroperitoneal hernia. Red arrow shows where the small bowel is penetrating through the retroperitoneal space.

An obese caucasian (BMI 34) 54 year-old female with a history of multiple abdominal surgeries including cholecystectomy, hysterectomy, gastric bypass, and most recently an anterior retroperitoneal spinal exposure for a spine fusion of L4-S1 presented to the emergency room with a recurrent small bowel obstruction. Patient had no significant past medical history. She had 2 previous obstructions within 3 months of her spinal fusion, both of which had been treated conservatively and resolved. However, on this admission, 6 months from the initial anterior lumbar exposure, conservative measures did not resolve the obstruction and the patient’s clinical status worsened with increasing abdominal pain, nausea and vomiting. A CT scan was obtained and demonstrated a small bowel obstruction similar to previous images seen at 2 months postoperatively, which appeared to be due to an internal hernia (See Fig. 1).

The patient was taken to the OR for exploratory laparotomy. Intraoperatively, the patient was found to have multiple loops of small bowel herniated through a small defect in the peritoneum. While the peritoneal defect did not appear to be the source of the obstruction, the small bowel was severely adherent to the left psoas muscle within the retroperitoneum, resulting in torsion and obstruction. A large incision was made into the retroperitoneal space and the small bowel was reduced following extensive sharp enterolysis. Postoperatively the patient had a prolonged hospitalization of 3 weeks due to several complications including an intra-abdominal abscess and a wound site infection.

## 3 Discussion

After a thorough search of the literature we were unable to find any reports of a symptomatic retroperitoneal hernia resulting in a small bowel obstruction as a complication of an anterior retroperitoneal spinal exposure.

Based on intraoperative findings, we feel that the retroperitoneal hernia was directly related to the anterior lumbar spine exposure and presumed secondary to peritoneal disruption during spine access, in which peritoneal disruption is a well-reported phenomenon. The anterior exposure was completed by a vascular surgeon with known experience in anterior exposures. In retrospective review of his operative report, the case was straightforward and he did not mention any issues with her anatomy due to previous open abdominal surgeries. There was mention of a peritoneal defect noted during the spine exposure procedure, with attempts to primarily close the defect made at that time.

Although the patient had several other abdominal surgeries, which might predispose her to an adhesive bowel obstruction, none of them violated the retroperitoneal space. There was clearly a peritoneal defect noted at the time of laparotomy through which the small bowel herniated through. We hypothesize that the small bowel herniated through the peritoneal defect and came in direct contact with a highly pro-adhesive environment. The amount of exposed muscle and soft tissue within the retroperitoneal space, in conjunction with inflammation and to some degree the hematoma from the initial spine exposure created an extensive raw surface area for the small bowel to adhere to. Literature suggests peritoneal tears should be repaired immediately to prevent enlargement [6], but does not directly address the phenomenon we describe here.

Upon reviewing the patient’s CT scans retrospectively, it is now clear that the retroperitoneal hernia was present on previous admissions and was likely the cause of her recurrent bowel obstructions. Due to the very rare nature of this hernia, the exact etiology was likely overlooked and since the patient was managed successfully with conservative treatment, no further intervention was pursued. Although the hernia was ultimately identified at the time of laparotomy and the patient recovered completely, she had a prolonged hospital course due to her complications following that procedure, which might have been negated had the initial peritoneal disruption not occurred or been fully repaired.

## 4 Conclusion

To our knowledge, this is the only report of a retroperitoneal hernia resulting in symptomatic small bowel obstruction requiring re-operation, as a complication of an anterior retroperitoneal spinal exposure. Although this patient’s complication was exceptionally rare, it underscores the critical importance of preserving the peritoneum as a biologic barrier during retroperitoneal spine exposures. While disruption of the peritoneum occurs not infrequently during these primarily retroperitoneal procedures, this case should serve as a cautionary tale and reinforce the need for identification and immediate primary repair of any peritoneal defects that may be created during this type of procedure.

## Conflicts of interest

No conflicts of interest to report.

## Sources of funding

No funding was received for this research.

## Ethical approval

This was a retrospective study; we are just reporting the outcome of the patient’s case with her consent. There was no interaction with patients for this research.

## Consent

Patient consented to the publication of this case.

## Author contribution

Colosimo – data collection, writing the paper

Ismail – editor

Schoeff - writing the paper, editor

Geiger - data collection

Lundy - data collection, writing the paper, editor

## Registration of research studies

This was a retrospective study; we are just reporting the outcome of the patient’s case with her consent. There was no interaction with patients for this research.

## Guarantor

Christina Colosimo.

## Provenance and peer review

Not commissioned, externally peer reviewed.
